# Acute social isolation and regrouping cause short- and long-term molecular changes in the rat medial amygdala

**DOI:** 10.1038/s41380-021-01342-4

**Published:** 2021-10-14

**Authors:** Danit Lavenda-Grosberg, Maya Lalzar, Noam Leser, Aseel Yaseen, Assaf Malik, Mouna Maroun, Liza Barki-Harrington, Shlomo Wagner

**Affiliations:** 1 Sagol Department of Neurobiology, Faculty of Natural Sciences, University of Haifa, Haifa, Israel; 2 Bioinformatics Service Unit, Faculty of Natural Sciences, University of Haifa, Haifa, Israel; 3 Department of Human Biology, Faculty of Natural Sciences, University of Haifa, Haifa, Israel

**Keywords:** Psychiatric disorders, Neuroscience, Molecular biology

## Abstract

Social isolation poses a severe mental and physiological burden on humans. Most animal models that investigate this effect are based on prolonged isolation, which does not mimic the milder conditions experienced by people in the real world. We show that in adult male rats, acute social isolation causes social memory loss. This memory loss is accompanied by significant changes in the expression of specific mRNAs and proteins in the medial amygdala, a brain structure that is crucial for social memory. These changes particularly involve the neurotrophic signaling and axon guidance pathways that are associated with neuronal network remodeling. Upon regrouping, memory returns, and most molecular changes are reversed within hours. However, the expression of some genes, especially those associated with neurodegenerative diseases remain modified for at least a day longer. These results suggest that acute social isolation and rapid resocialization, as experienced by millions during the COVID-19 pandemic, are sufficient to induce significant changes to neuronal networks, some of which may be pathological.

## Introduction

The survival and success of individuals of gregarious mammalian species depend on their ability to form social interactions [1]. These individuals usually habitat in social structures and their wellbeing and survival are threatened by social isolation [2–4]. It is therefore not surprising that in humans, among the most social species on earth [5, 6], real or perceived social isolation is associated with compromised health and increased mortality rates [7, 8]. Social isolation also affects mental health [9] and is associates with higher rates of Alzheimer’s disease (AD) [10–12]. Actual and perceived loneliness are very common among modern societies [13, 14], with as many as 40% of adults over 65 years of age reporting being lonely at least sometimes [15–17]. Moreover, the recent ongoing COVID-19 pandemic has forced millions around the world into repeated social isolation periods ranging from several weeks to months [18], a situation which may reoccur in future pandemics.

Whereas the psychological effects of social isolation are widely studied, there is much less information about its short- and long-term effects on brain circuitry and activity. While exploration of this important issue in humans presents a major obstacle, mice and rats are optimal models for deciphering the brain mechanisms that are affected by social isolation [19, 20]. Indeed, the consequences of extended social isolation periods were widely explored in these animal models (Reviewed in [21]). However, there is much less information regarding the consequences of short periods of social isolation (acute social isolation herein) on the brain [22, 23]. We and others have previously reported that just several days of social isolation significantly impair social recognition memory (SRM) of laboratory rats and mice [20]. Regrouping of the animals after isolation reversed the behavioral effect, suggesting that the brain circuitry has the ability to adapt to changing social conditions [24]. SRM is linked to the medial nucleus of the amygdala (MeA) [25–27], an area that is also involved in human social behavior [28]. Therefore, the purpose of the present study was to identify the molecular and biochemical signatures of the changes that occur in the MeA of adult male rats during acute social isolation and at different time points during regrouping.

## Results

### Social isolation and regrouping differentially affect gene expression in the MeA

**Fig. 1:**
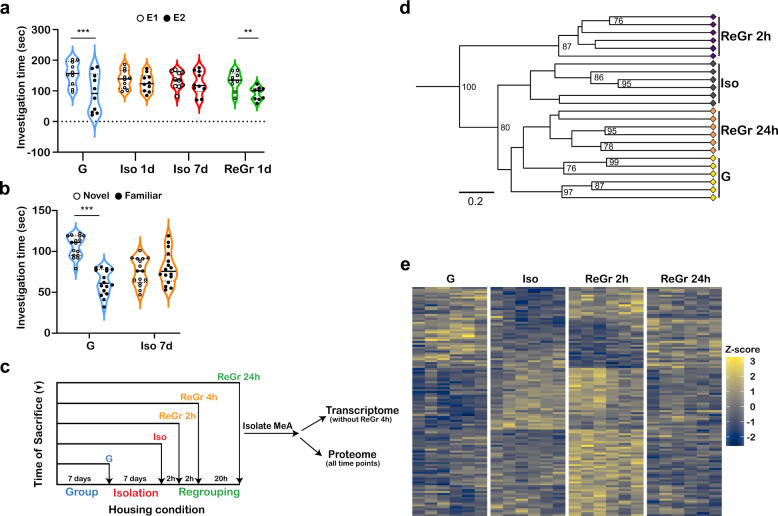
Social isolation and regrouping differentially affect gene expression in the medial amygdala (MeA). **a** Social recognition memory (SRM), demonstrated by a reduction in investigation time between two consecutive 5-min encounters (E1, E2) of the subject rat with the same social stimulus, using a 120 min inter-encounter interval. Note the rapid loss of memory one and seven days following isolation (Iso 1d. Iso 7d), compared to group housing (G) and its rapid restoration 1d following regrouping (ReGr 1d). **p < 0.01, ***p < 0.001, paired *t*-test following main effect in 2-way repeated ANOVA. Horizontal lines represent median values. **b** Social novelty preference of G or Iso 7d subjects (*n* = 16 per group) towards novel or familiar conspecifics, 24 h after a 60 min encounter with the familiar conspecific. ***p < 0.001, paired *t*-test following main effect in 2-way repeated ANOVA. Horizontal lines represent median values. **c** Experimental design of omic experiments. Animals were divided into four experimental conditions: grouped (G), isolated for seven days (Iso), isolated for seven days and then regrouped for two hours (ReGr 2 h), regrouped for 24 h following isolation (ReGr 24 h). The proteomics experiment had an additional group, of animals regrouped for 4 h after isolation (ReGr 4 h). Following decapitation, MeA samples were extracted and processed for either transcriptomic or proteomic analyses. **d** Hierarchical clustering according to transcription level of 119 differentially expressed (DE) genes, for the four different experimental conditions, based on Euclidean distance matrix of log-transformed FPKM values. Values at the nodes indicate bootstrap values (as percent of 1000 replications). Bootstrap values below 75% were omitted. **e** Heat-maps of a Z-score analysis of the DE genes shown in **c**, for each animal in the four experimental groups. Z-score was calculated according to the average of all 24 samples (six animals in four groups) per gene.

In order to study the effect of acute social isolation on the MeA, we used a behavioral SRM model, whereby we measured the time spent by an adult male Sprague Dawley rat (*n* = 10) housed in group housing (G) in exploring a same-sex juvenile upon a second encounter (E2). If SRM is formed, the time spent examining the stimulus is significantly reduced compared to the first encounter with the same individual (E1), which took place 2 h earlier. The results presented in Fig. 1a are in accordance with the ones we previously obtained [24], and show severe impairment of SRM in animals that experienced social isolation for one day (Iso 1d), which lasts throughout seven days of isolation (Iso 7d). One day after returning the animals to group housing (ReGr 1d), SRM is fully restored (2-way repeated ANOVA - encounter × condition: *F*_(3,27)_ = 5.368, *P* = 0.005; *post hoc* paired *t*-test - G:t_9_ = 6.345, *p* < 0.001; ReGr 1d:t_9_ = 4.088, *P* = 0.007).

Previous studies reported that social isolation might increase the motivation of rats for social interactions [29, 30]. To confirm that our results reflect impaired SRM rather than changes in general motivation for social interactions, we conducted an additional set of social novelty preference experiments, using G and Iso 7d animals. In this paradigm of long-term SRM [27], subject animals were first exposed to a conspecific for 60 min. Twenty four hours later, the subjects were exposed simultaneously to the same conspecific (familiar), as well as a new conspecific (novel), and the time dedicated to their investigation was measured. Statistical analysis revealed a highly significant interaction between individuals (novel vs. familiar) and housing conditions (G vs. Iso 7d) (2-way repeated ANOVA - *F*_(1,30)_ = 62.57, *P* < 0.0001). *Post hoc* analyses revealed that G subjects show significant preference for the novel individual (paired *t*-test, *t*_15_ = 7.586, *P* < 0.0001), thus exhibiting intact SRM. In contrast, Iso 7d animals were unable to discriminate between the novel and familiar conspecifics (*t*_15_ = 0.751, *P* = 0.703), thus confirming that seven days of social isolation impairs SRM (Fig. 1b). No difference was found between the two conditions in the total time the animals spent exploring both stimuli together (*t*-test, *t*_30_ = 1.382, *P* = 0.177), suggesting no differences in the motivation for social interaction between the two groups.

In order to characterize the transcriptional changes that occur in the rat MeA in response to acute social isolation and regrouping, we performed an RNA-Seq analysis where animals were assigned one of the following conditions: (1) Grouped (G); (2) Isolated (Iso) that were isolated for seven days; (3) Regrouped for 2 h (ReGr 2 h) that were regrouped for 2 h following seven days of isolation, and (4) Animals regrouped for 24 h after isolation (ReGr 24 h) (Fig. 1c). The rats used for this analysis did not perform any social behavioral test and were not exposed to a juvenile social stimulus prior to being sacrificed.

The transcriptome analysis identified 16,227 genes (Supplementary Data file 1), 119 of which showed differential expression (DE herein) among the distinct groups (FDR threshold *P* < 0.05, FPKM > 0.3, log_2_FC > ±0.2; Supplementary Fig. 1). As depicted in Fig. 1d, hierarchical clustering of the DE gene set (Euclidean distances between samples log-transformed FPKM, 1000 bootstrap steps) presented similar gene expression patterns between the G and ReGr 24 h animals, suggesting that the mRNA levels of most genes return to baseline 24 h after regrouping. In contrast, gene expression in the Iso and ReGr 2 h groups, showed marked differences compared to the G animals (Fig. 1d). This phenomenon, also apparent in the heat-map of the same gene set (Fig. 1e), suggests that the MeA transcriptome responds rapidly and dynamically to the acute changes in social conditions.

In order to validate the RNA-Seq results, we selected six of the 119 DE genes and used qPCR to measure their mRNA levels in the same samples that were used for the RNA-Seq analysis. We selected three genes that were significantly elevated (*FosB*, *Hspa5*, *Bdnf*) and three that were significantly reduced (*Slit2*, *Mmp14* and *Crhbp*) between Iso and ReGr 2 h. We also preferred genes that showed a particularly high fold-change (Supplementary Fig. 1a) and were associated with inter-cellular signaling. All genes exhibited a significant correlation (Pearson’s correlation, *P* < 0.05) between the qPCR and RNA-Seq results (Supplementary Fig. 1b, Supplementary Data File 2.

A previous study reported that odor-enriched environment (OEE) rescues SRM in mice isolated for seven days [31]. To examine if a similar effect occurs in rats, we compared the SRM of G rats and Iso 7d rats that were kept in OEE. While there was a borderline significant interaction between encounter and condition (Mixed model ANOVA - encounter × condition: *F*_(1,22)_ = 4.338, *P* = 0.049), *post hoc* analysis revealed that OEE rescued SRM in acutely isolated rats, as both groups showed a significantly reduced investigation time in E2 compared to E1 (*post hoc* paired *t*-test - G:*t*_10_ = 5.131, *P* < 0.0001; Iso 7d:*t*_12_ = 4.836, *P* < 0.0001; Supplementary Fig. 2a). To examine if this OEE-mediated rescue is also reflected in transcriptional changes, we examined one of the above genes, *Crhbp*, and found no significant difference between the groups (*t*-test, *t*_10_ = 1.475, *P* = 0.171; Supplementary Fig. 2b, Supplementary Data File 2), suggesting that besides SRM, OEE rescues at least some of the transcriptional changes caused by acute isolation.

### Social isolation and regrouping affect neuronal network remodeling

**Fig. 2:**
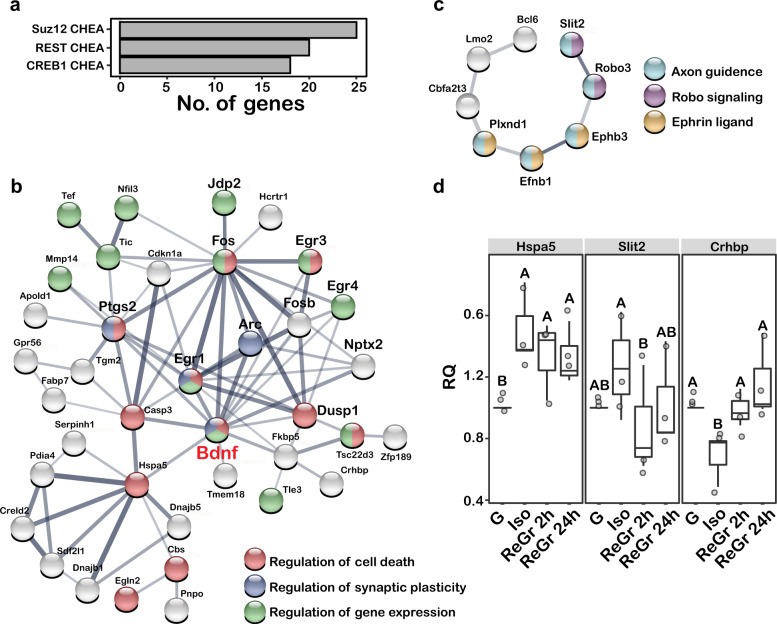
Differentially expressed (DE) genes are enriched in BDNF signaling and axon guidance pathways. **a** Functional pathways enriched among DE gene set, queried against the ENCODE CHhA consensus transcription factor (TF) targets from Chip-X database. Presented are TF target sets for which FDR adjusted *P* < 0.05. **b** The largest network revealed by STRING analysis of DE gene set showing a minimum interaction score of medium confidence (0.4). Line thickness indicates the strength of data support. Note that this network is enriched in immediate-early genes (in bold) and genes associated with regulation of cell death, synaptic plasticity and gene expression (color coded according to association). As in **c**, for the second largest network revealed by STRING analysis. Note that this network is enriched in genes associated with axon guidance, Robo and Ephrin signaling (color coded according to association). **d** Normalized RQ values of qPCR analysis of three independent sample sets (Exp-1,2,3,) for three genes representing the networks shown in **b** (Hfspa5, Crhbp) and **c** (Slit2). Letters represent statistically significant groups.

We next used Enrichr [32] to identify the pathways that are enriched among the DE genes (Supplementary Data File 1). This analysis revealed a significant enrichment of genes belonging to the Brain-derived neurotrophic factor (Bdnf) signaling pathway (Benjamini-Hochberg, adj. *P* < 0.001). Compared to the Iso group, the mRNA levels of *Bdnf* itself were significantly upregulated in the ReGr 2 h and ReGr 24 h animals (log2FC = 0.5, *P* = 0.001 and log2FC = 0.49, *P* = 0.004, respectively). Additional enrichment was found for targets of three transcription factors: Suz12, Rest and Creb1 (Fig. 2a, adj. *P* = 0.002, 0.003 and 0.002, respectively), which are associated with brain development [33–37] and memory formation [38, 39].

Application of the STRING database [40] on the DE genes revealed several enriched networks, the largest of which included genes associated with Bdnf signaling, as well as multiple immediate-early genes (Fig. 2b). Additional enriched gene ontologies (GO) were those associated with regulation of cell death (GO: 0010941, FDR adj. *P* = 0.009), regulation of synaptic plasticity (GO: 0048167, FDR adj. *P* = 0.013) and regulation of gene expression (GO: 0010468, FDR adj. *P* = 0.010). The next largest network was that of genes associated with axon guidance including *Slit2*, *Robo3*, *Ephb3*, *Efnb1* and others (Fig. 2c).

We next selected the following genes from each network for verification by qPCR: *Hspa5* and *Crhbp* from the Bdnf network, and *Slit2* from the axon guidance network. For these experiments, we used the original sample set from the RNA-Seq analysis (Exp-1) and two additional sets obtained from independent experiments (Exp-2, Exp-3). As shown in Fig. 2d, the qPCR experiments confirmed the data obtained by the transcriptome analysis for all genes. *Hspa5*, also known as *BiP* or *GRP78*, is a heat-shock protein who’s function is central to ER-associated protein degradation and linked to autophagy/apoptosis processes [41]. The mRNA levels of this gene were consistently higher during isolation (Kruskal–Wallis – H3 = 16.16, *P* = 0.001; Fig. 2d, Supplementary Fig. 3). Similarly, mRNA levels of Slit2, a secreted ligand of Robo receptors that play a central role in axon guidance [42, 43], were also markedly elevated in Iso animals and significantly dropped after just two hours of regrouping (Kruskal–Wallis – H_3_ = 8.74, *P* = 0.033; Fig. 2d, Supplementary Fig. 3). Finally, the levels of *Crhbp*, a brain-wide abundant secreted glycoprotein thought to regulate stress [44], were significantly reduced during isolation and returned to baseline two hours after regrouping (Kruskal–Wallis – H_3_ = 16.882, *P* < 0.05; Fig. 2d, Supplementary Fig. 3). Validation of the RNA-Seq data using independent sample sets strengthens our observation that acute isolation and regrouping are accompanied by changes in a specific cohort of signature genes that are associated with neuronal network remodeling such as neurotrophic signaling, synaptic plasticity and axon guidance.

**Fig. 3:**
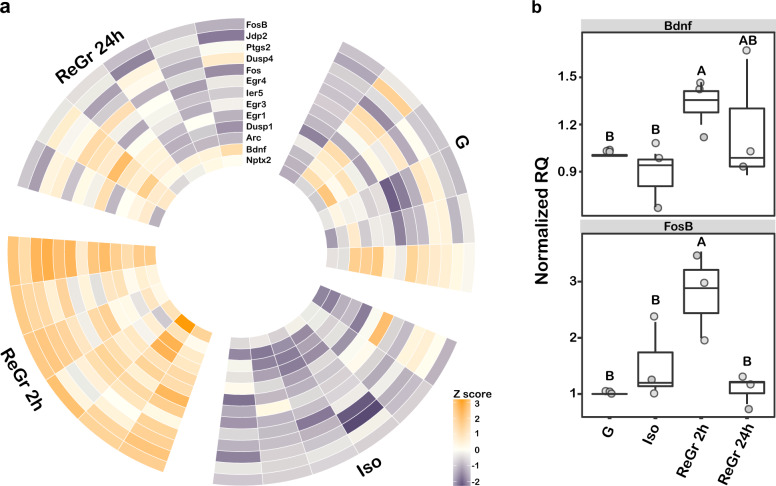
Regrouping caused a transient elevation in mRNAs of immediate-early genes (IEGs). **a** A Heat-map of Z-score analysis of 13 IEGs that were differentially expressed between the various conditions. Note that mRNA level of most genes increased two hours after regrouping and returned to baseline 24 h later. **b** Normalized RQ values of the three independent sample sets (Exp-1,2,3,) for *Bdnf* mRNA levels in the MeA (top) and *Fosb* (bottom). Letters represent statistically significant groups.

Among the largest network of the DE genes in the STRING analysis, we identified a group of nine immediate-early genes (IEGs, Fig. 2b). Since IEGs are not listed as an ontology, we assembled our own list (Supplementary Data File 1) and used the Chi square test with Yates correction to determine whether they are significantly enriched among the 119 DE genes. This analysis revealed that 11 of the 25 IEGs on our list (44%, χ^2^ = 403.7, *P* < 0.0001) were significantly upregulated in the ReGr 2 h compared to the Iso animals (Fig. 3a). To validate these results, we performed qPCR on two genes, *Bdnf* and *FosB*, which are common to both the IEGs and the Bdnf signaling pathway. In all three independent experiments (Supplementary Data File 2), the mRNA levels of both genes were significantly higher in the ReGr 2 h compared to both Iso and G animals, with no significant changes between the latter two (Fig. 3b, Supplementary Fig. 3). These results further validate our RNA-Seq analysis and confirm that acute isolation and regrouping lead to induction of specific IEGs and Bdnf signaling-associated genes.

### Social isolation and regrouping modify protein expression

To determine if acute isolation and regrouping affect protein expression, we prepared another set of MeA samples under the exact experimental conditions described above. Given the significant changes in mRNA expression after 2 h of regrouping, we reasoned that changes in protein levels may follow, and therefore added another group of animals sacrificed four hours after regrouping (ReGr 4 h) (Fig. 1c).

The proteomic analysis identified 4968 expressed proteins, 4767 of which (~96%) were also found by the RNA-Seq analysis (Supplementary Data File 3). Compared to G animals, 95 proteins were differentially expressed in the Iso group, 77 of which (80%) returned to their initial levels in the ReGr 24 h animals. This suggests that most acute isolation-induced changes in protein levels are transient. However, the levels of 18 proteins remained altered, suggesting that acute isolation may leave a long-term signature. Functional enrichment analysis of these 18 proteins identified the chaperonin-mediated protein folding cluster (R-HAS-390466, adj. *P* = 0.003), including subunits 1 and 4 of Prefoldin, a driver of AD [45]. Among these proteins were also C1qb, Bag5 and Sv2c, which are associated with AD [46–48]. Three additional proteins (Arhgef7, Kalrn, Rgma) belong to the axon guidance pathway (R-HAS-422475, adj. *P* = 0.044) and three others (Golga2, Snx32, Wdr41) belong to autophagy regulation (GO: 0010506, adj. *P* = 0.059).

**Fig. 4:**
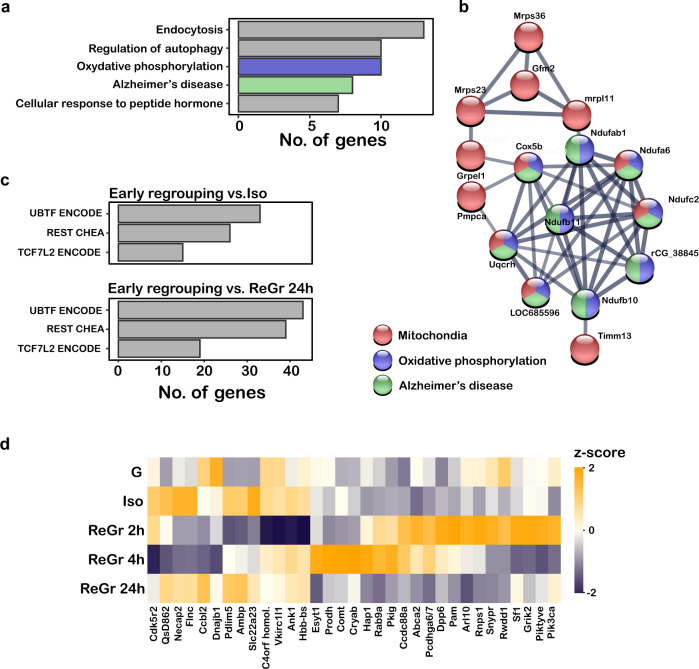
Proteomic changes in the MeA following isolation and regrouping. **a** Functional pathways enriched among the 183 proteins that are differentially expressed (adj. *P* < 0.05) between G and ReGr 24 h animals, queried against the KEGG and GO databases. **b** A network revealed by STRING analysis of the same set of 183 DE proteins as in **a**. Line thickness indicates the strength of data support. Note that this network is enriched in proteins associated with the mitochondria, oxidative phosphorylation and Alzheimer’s disease (color coded according to association). **c** Functional pathways enriched among 194 proteins that are differentially expressed between Early regrouping (ReGr 2 h and ReGr 4 h) and Iso animals (upper panel) or ReGr 24 h animals (lower panel), queried against the ENCODE CHhA consensus transcription factor (TF) targets from Chip-X database. Presented are TF target sets for which FDR adjusted *P* < 0.05. **d** Heat-map of Z-score analysis of the 33 of the 40 proteins that were differentially expressed in Early regrouping compared to both Iso and ReGr 24 h animals. These 33 proteins showed opposite trends under the different conditions.

The levels of 183 proteins were markedly changed between the ReGr 24 h compared to the G animals, suggesting the long-term effects that are the result of the regrouping process itself. Among these were proteins associated with endocytosis (KEGG: mmu04144. adj. *P* = 0.0003), oxidative phosphorylation (KEGG: mmu00190, adj. *P* = 0.0002), neurodegenerative diseases (KEGG: mmu05010, adj. *P* = 0.0004) and regulation of autophagy (GO: 0010506, adj. *P* = 0.015) (Fig. 4a, Supplementary Data file 3). STRING analysis revealed a dense network of proteins, the core of which were associated with oxidative phosphorylation and mitochondrial electron transport chain, which overlapped with genes associated with AD (Fig. 4b).

To characterize the immediate effects of regrouping, we identified the proteins that changed between the Iso and ReGr 2 h or ReGr 4 h animals (Early Regrouping, herein). The expression of 194 proteins was significantly different between Iso and Early regrouping, 98 were unique to ReGr 2 h, 82 to ReGr 4 h and 14 were common to both, suggesting the existence of highly dynamic changes in protein expression within a very short time frame after regrouping. Functional analysis of the DE proteins in the Early Regrouping samples revealed enrichment of several inter-cellular signaling cascades, including the extracellular signal-regulated kinases (ERK) (BIOCARTA: M6355, adj. *P* = 0.012), and growth hormone signaling pathways (BIOCARTA: M9043, adj. *P* = 0.023) (Supplementary Data file 3). Analysis of transcription factor targets in the Early Regrouping animals revealed enrichment of targets of several transcription factors including Ubtf, Tcf7l2 and Rest, all of which are related to neurogenesis and brain development [33, 49, 50]. Targets of these three transcription factors were also enriched among the 225 proteins that were differentially expressed between Early Regrouping and ReGr 24 h animals (Fig. 4c). Notably, Rest targets were also enriched in the transcriptome analysis (Fig. 2a). These results strongly support the involvement of Ubtf, Tcf7l2 and Rest in the molecular changes that take place in the MeA immediately after regrouping.

To further explore the dynamics in protein expression following regrouping, we identified 40 proteins that changed in Early Regrouping compared to both Iso and ReGr 24 h animals. Remarkably, 33 of these proteins (83%) showed opposite trends between the early and late regrouping events (Supplementary Data File 3). These proteins represent processes that are strongly affected immediately after regrouping and return to baseline 24 h later, thus supporting the functionality of the observed proteomic changes (Fig. 4d).

### Social isolation and regrouping cause dynamic molecular changes

A comparison between the proteomic and transcriptomic data yielded only eight differentially expressed genes that were common to both analyses (*Crhbp*, *Synpr*, *Adgrg1*, *Dnajb1*, *Fabp7*, *Fam107a*, *Mblac2* and *Slc20a2*). We therefore reasoned that rather than overlapping, the two analyses are complementary. To examine the overall molecular effect of isolation and regrouping, we combined the results of both analyses into a single list of 812 DE genes/proteins. In terms of signal transduction pathways, this analysis revealed enrichment in two main non-overlapping signaling pathways, namely Bdnf and Pdgf (BioPlanet 2019, adj. *P* < 0.0001 for both). Among the different cellular processes that were enriched, the most general ones were endocytosis (KEGG: mmu04144, adj. *P* = 0.009), autophagy (KEGG: mmu04140, adj. *P* = 0.016) and axon guidance (R-HAS-422475, adj. *P* = 0.015). In terms of disease, we found a borderline enrichment in mitochondrial dysfunction associated with neurodegenerative diseases such as AD, Huntington’s disease and Amyotrophic Lateral Sclerosis (Elsevier pathway collection, adj. *P* = 0.076).

**Fig. 5:**
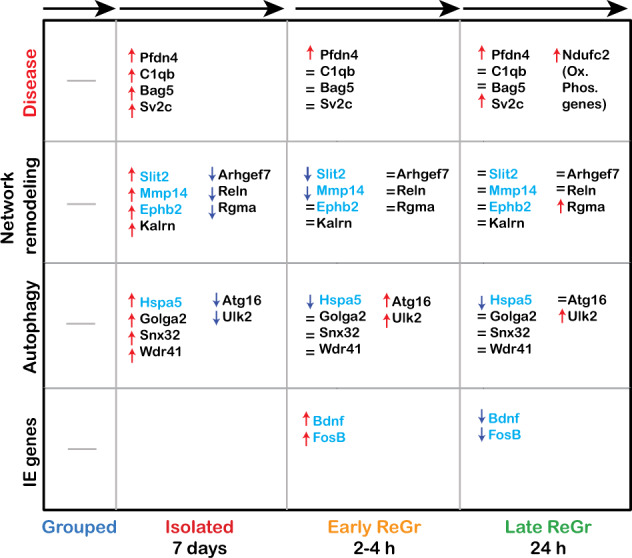
Functional pathways affected by the various stages of social isolation and regrouping. Overall view of the dynamic molecular changes in the MeA, caused by isolation and regrouping. Shown are four categories: IEGs, Autophagy, Neuronal network remodeling and Disease. Transcriptomic data is marked in light blue and proteomic data - in black. The information in each box refers to change from the previous condition. (=) means no change.

To provide an overall view on the dynamics of the molecular changes that occur during isolation and regrouping, we defined four categories of processes that were affected: IEGs (representing rapid signaling events), Autophagy (representing synaptic processes), Network remodeling (representing axon guidance and cell adhesion processes) and Disease. As shown in Fig. 5, IEG levels were increased during Early Regrouping and returned to baseline within 24 h. In contrast, cellular autophagy processes showed a mixed response; while some genes (*Golaga2*, *Snx32* and *Wdr42*) were upregulated during isolation and remained elevated after regrouping, *Ulk2* and *Atg16L1* that control autophagosome initiation and nucleation steps [51] were downregulated during isolation and upregulated during regrouping. Interestingly, *Hspa5*, exhibited opposite trends, with upregulation during isolation and reduction immediately after regrouping. Similarly, network remodeling processes showed elevation in some genes associated with axon guidance during isolation, the levels of which dropped following regrouping (e.g., *Slit2*, *Mmp14*). In contrast, other proteins were downregulated during isolation some of which remained low after regrouping (e.g., Arhgef7 and Reln), while others returned to baseline (Rgma). Finally, several proteins that are associated with neurodegenerative diseases were upregulated either during isolation (Pfn4, C1qb, Bag5 and Sv2c) or following regrouping (oxidative phosphorylation-associated genes such as Ndufc2). Together these data suggest that acute social isolation and regrouping induces significant transient changes in the neuronal network of the MeA while leaving a longer lasting molecular signature associated with neurodegenerative diseases.

## Discussion

We have previously demonstrated a severe impairment in SRM of adult rats within just 24 h of social isolation, which is rapidly reversed following regrouping [24]. SRM was previously shown to be associated with de novo protein synthesis [52]. Here, we studied the molecular changes that occur in the MeA during acute social isolation and regrouping. Our main findings were that each stage has its own molecular signature, and that while most changes are reversible within one day of regrouping, some last longer, particularly those associated with neurodegenerative disorders.

Most studies that explore the effects of social isolation use models of extended isolation (>1 month) that begin immediately after weaning. Such harsh isolation conditions cause various abnormalities [21, 23]. However, they do not mimic the milder types of real or perceived social isolation that are commonly experienced by people [53]. Moreover, these models do not recapitulate much shorter periods of social isolation, such as experienced by millions due to the COVID-19 pandemic [54]. Our model, which better reflects the aforementioned conditions, demonstrates that acute social isolation induces rapid behavioral and molecular effects on the brain. Furthermore, while the SRM is seemingly restored with respect to behavior, acute isolation leaves a long-term molecular signature that may affect the individual later in life. Although our results may be limited to molecular changes and do not reflect anatomical modifications, they suggest the occurrence of neuronal network remodeling in the rat MeA in response to acute social deprivation and regrouping.

Two limitations of the study emerge from the time points used for sampling the molecular changes. First, the lack of overlap in DE genes and proteins may stem from the fact that many genes take longer than four hours to express [55]. Second, since we did not investigate a time point that is longer than 24 h, we may have missed some long-term changes that occur later. Nonetheless, our study describes the general dynamics of the molecular changes that occur in the MeA during acute isolation and regrouping, which may leave a persistent mark.

The MeA was previously shown to be involved in molecular processes crucial for SRM formation in both rats and mice [25–27]. As such, it receives social-specific chemosensory information that is necessary for SRM formation [56, 57]. During acute isolation, these signals are markedly reduced, and upon regrouping, become abundant and strong. These fluctuations may explain the vast molecular changes following isolation and regrouping. These changes suggest substantial neuronal network remodeling, which may be analogs to reported anatomical changes in sensory cortical areas following sensory deprivation in adulthood [58, 59]. Nevertheless, we cannot exclude the possibility that these changes may involve additional aspects of social behavior, such as altered social hierarchy due to social isolation and regrouping.

Electrophysiological changes following 24 h of social isolation were reported in other brain areas. Notably, Matthews et al. [60] showed in mice that acute social isolation raises the neuronal activity in dopaminergic neurons of the dorsal raphe nucleus, which enhance the motivation of the animal to interact with social, but not object stimuli. Our study did not examine social motivation per se, but rather showed that acute isolation directly affects SRM (Fig. 1b). Although we cannot exclude the possibility that social motivation undergoes parallel changes that are separate from those of social memory, our results do not support this possibility for two reasons. First, as depicted in Fig. 1a, there is no change in the investigation time in the first encounter (E1) between grouped, isolated and regrouped animals. Second, the total investigation time of both familiar and novel conspecifics in the social preference experiment was the same in isolated and grouped animals (Fig. 1b). A possible explanation for the differences between the study by Matthews et al. and ours is the use of different models systems (mice vs. rats). We have recently shown that compared to mice, rats present with higher and more immediate motivation for social interactions [61]. Accordingly, a pervious study found that changes in the social motivation of acutely isolated rats are restricted to young age [62].

George et al. [63] examined changes in gene expression 1–2 days after social isolation, in the caudomedial forebrain of Zebra finches [64]. The authors reported molecular changes that are similar to ours, with enrichment in the neurotrophin signaling and axon guidance pathways. Notably, several differentially expressed genes were found to overlap between the two studies, including *Egr1*, *Dusp4*, *Bdnf* and *Fkbp5*, the latter of which is strongly associated with stress response [65, 66]. These results may reflect an evolutionarily conserved mechanism of brain molecular responses to social isolation.

Interestingly, our results suggest that many of the molecular changes occur after returning from isolation to group housing. These changes may be divided into two categories. The first category involves mostly *Creb1* targets (e.g., *FosB*, *Bdnf*) that are strongly elevated two hours following regrouping (Figs. 2 and 3). The second category, which is associated with network remodeling, may also be induced by IEGs associated with long-term plastic changes, (e.g *Arc*) [67]. This effect may be attributed to two additional transcription factors, Rest and Suz12, the downstream targets of which are enriched within the DE genes (Fig. 2). Rest targets were also enriched among the DE proteins. These transcription factors play significant roles in brain development and in neurodevelopmental disorders [34, 68]. Suz12 a component of the polycomb repressive complex 2 (PRC2), which has a well-established role in CNS development [69] and neurodegenerative diseases [70], specifically in Huntington’s disease [71]. Rest is another epigenetic factor, which besides its role in neurogenesis [72], is implicated in Huntington’s disease [73]. Importantly, Rest represses genes that promote cell death and AD and protects neurons from oxidative stress and amyloid β-protein toxicity [74]. AD and oxidative phosphorylation are ontologies that are enriched among the genes that showed long-term changes following social isolation and regrouping.

Multiple studies (reviewed by Cacioppo et al. [75]), link loneliness to cognitive decline, dementia and AD in elderly people. Our results reveal several possible mechanisms for this link. First, among proteins that changed significantly after 24 h of regrouping, we found enrichment in genes associated with oxidative phosphorylation and mitochondrial electron transport chain, a pathway strongly linked to AD and other types of neurodegeneration [76]. Interestingly, hippocampal administration of an antioxidant alleviate early cognitive deficits induced by social isolation in a genetic mouse model of AD [77]. Second, the changes we observed in genes associated with Bdnf signaling following isolation and regrouping may be linked to AD, as a recent large human study linked between social relationship measures, serum Bdnf levels, and the risk of stroke and dementia [78]. Finally, among the 18 genes that changed during isolation and did not return to baseline following regrouping, were two subunits of the prefoldin complex that is known to inhibit both Aβ fibril formation and α-synuclein aggregation [45].

Overall, our results support the “social homeostasis theory”, recently proposed by Mathews and Tye, according to which brain mechanisms adapt in order to maintain a stable level of social interactions [79]. Similar to other homeostatic systems, social hemostasis mechanisms are likely to involve behavioral, physiological and molecular adaptations. In accordance with these theories, our results suggest that social deprivation and a following overload of social stimuli, can induce opposite adaptations not only in neural activity of social behavior associated brain regions, but also in the structure of their neural networks. Thus, short periods of social isolation followed by resocialization, as experienced by million during the current COVID-19 pandemic, may have much more significant implications on brain processes than is currently appreciated.

## Materials and methods

### Animals

Sprague Dawley male rats (adult 7–8 week, 225–249 g or juvenile 3 week, 30–35 g) as well as Wistar-Hola male rats were purchased from Envigo (Rehovot, Israel) and maintained under a 12-h light/dark cycle, at a temperature of 22 ± 2 °C, with food and water available *ad libitum*. All experiments were performed according to the National Institutes of Health guide for the care and use of laboratory animals, and approved by the Institutional Animal Care and Use Committee (IACUC) of the University of Haifa.

### Behavioral experiments

#### Social recognition memory (SRM)

The SRM test was conducted as previously described [24]. See Supplementary Methods for further details.

#### Social novelty preference (SNP)

SNP experiments were carried out as previously described [27]. See Supplementary Methods for further details.

#### Odor-enriched environment (OEE)

OEE was established through the addition of commercial fruit odors that are regularly used in the cosmetics and food industry [80] to the home cages of the socially isolated rats. The odors were renewed daily throughout the seven days of isolation, by moving each animal every day to a new cage with a new fresh odor absorbed in its bedding. The mixture of clean bedding (200 g) and odor (30 µ of pure essences in 15 ml) for each cage was prepared daily, 4 min before exposure.

### Brain sampling and processing

All rats were decapitated and their brains immediately removed, immediately frozen on dry ice and stored at −80 °C for until further processing. The MeA was bilaterally punched from 400 μm slices using a 1 mm punching needle in a cryostat at −20 °C. Samples were then placed in Eppendorf tubes and stored at −80 °C for until further processing.

RNA extraction for RNA-Seq analysis as was carried out using RNeasy Lipid Tissue Mini Kit (Qiagen), according to the manufacturer’s instructions. Concentration of RNA was determined using Nanodrop-1000 (Thermo Scientific). mRNA libraries were prepared using TruSeq RNA Library Prep Kit v2 (Illumina) according to the manufacturer’s protocol, and sequenced on HiSeq 2500 (Illumina) 50 bp single read run. Sequences were obtained in fastq format from the TGC Sequencing and Bioinformatics Services, Technion Genome Center, Haifa, Israel.

### RNA-Seq data analysis

Sequences were de-multiplexed by sample based on sample unique barcode. Reads were adapter-trimmed using cutadapt 1.15, then low-quality regions were removed with Trimmomatic 0.3. The filtered dataset was inspected in Fastqc. Illumina reads were mapped to the Rattus_norvegicus.Rnor_6.0.90 assembly downloaded from Ensembl using Star v2.5 (Dobin, et al., 2013). Differential expression analysis was conducted using Bioconductor EdgeR (McCarthy, Chen, & Smyth, 2012) based on RNA-Seq raw read counts per gene. In EdgeR, within-samples normalization is performed using the TMM algorithm by default. In addition, EdgeR between-samples normalization accounts for differences in library size. Subsequently, the program produces a gene-specific biological variation estimate based on an Empirical Bayes method. Based on this per-gene biological variation estimate, the program allows fitting a specific Generalized Linear Model (GLM) to the gene data, and tests for differential expression. EdgeR GLM is based on a negative binomial distribution function by default. Here, in the GLM, we considered the additive effect of Batch and the Group factors. Expression was considered significantly different for genes with FDR adjusted *P* value < 0.05. We further filtered out genes with considerable variation among batches. Gene set enrichment testing was performed with Enrichr [32] quarrying differentially expressed genes against BioPlanet, ENCODE and ChEA consensus transcription factors, GO, KEGG and Reactome databases (accessed May, 2021). Gene association networks were calculated for the differentially expressed gene set using STRING [40] accessed on May 2021. Minimum interaction score was set at 0.4 (medium confidence), excluding interactions from text mining or databases. Line thickness indicates the strength of data support.

### Quantitative PCR

For the two repeated experiments done for verification purposes (Exp-2, Exp-3), RNA extraction was done with Tri-reagent solution (Sigma). Each reaction contained PerfeCTa SYBR green FastMix (Quanta Bioscinces), 10 ng of cDNA (synthesized using the qScript cDNA Synthesis Kit, QuntaBio) and 10 μM of each primer (Supplementary Data File 2). SYBR RT PCR reactions were conducted on a StepOne plus Real-Time PCR instrument (Applied Biosystems). See Supplementary Methods for further details.

### Protein extraction and proteomic analysis

Proteomics sequencing was performed by the de Botton Institute of Protein Profiling at the Weizmann Institute for Science (Rehovot, Israel). The proteomic analysis was performed on an independent cohort of animals (*n* = 30, *n* = 6 per group) (see Study design section).

Immediately following MeA removal, 50 µl of lysis buffer (100 mM Tris pH 7.6, 5% SDS) were added to the microtubes, and samples were homogenized, centrifuged at 16,000 × *G* at 4 °C, and the lysates were kept at −80 °C until further processing. Lysates were subjected to a solution tryptic digestion using the S-Trap method (ProtiFi), followed by a desalting step. The resulting peptides were analyzed using nanoflow liquid chromatography (nanoAcquity) coupled with high-resolution, high mass accuracy mass spectrometry (HF). Each sample was analyzed by the instrument separately, in random order in the discovery mode.

### Statistical analysis

Statistical analyses were carried out with GraphPad Prism 7.04 software for Windows, SPSS v21.0 (IBM) and R (v4.1.0). Specific analyses and tests are described in each section. All *t*-tests were two-sided.

## Supplementary information

Supplementary information

Supplementary Methods

Supplementary Figures

Data File S1

Data File S2

Data File S3

## Data Availability

All data needed to evaluate the conclusions in the paper are present in the paper and/or the Supplementary Materials. The proteomic raw data are available by the ProteomeXchange Consortium via the PRIDE partner repository with the dataset identifier PXD026871. The transcriptomic raw data are available via NCBI SRA identifier PRJNA734478.
