# Supplementary material for: Acute social isolation and regrouping cause short- and long-term molecular changes in the rat medial amygdala
Source: Mol Psychiatry. 2021 Oct 14;27(2):886–95. doi: 10.1038/s41380-021-01342-4 (PMC8515782; doi:10.1038/s41380-021-01342-4)
Supplement: Supplementary file 1 — Supplementary information [file 41380_2021_1342_MOESM1_ESM.pdf]

Supplementary Information

Supplementary Methods. Detailed description of the methods

Supplementary Figures:

Supplementary Figure 1. Validation of RNA-seq results by qPCR in selected genes

Supplementary Figure 2. Odor-enriched environment (OEE) rescues SRM is isolated animals

Supplementary Figure 3. Real-time qPCR validation of transcriptomic changes in five genes, in the three independent MeA sample sets

Supplementary Data file 1. Transcriptomic information

Supplementary Data file 2. Real-time qPCR results

Supplementary Data file 3. Proteomic data
