# Supplementary material for: Acute social isolation and regrouping cause short- and long-term molecular changes in the rat medial amygdala
Source: Mol Psychiatry. 2021 Oct 14;27(2):886–95. doi: 10.1038/s41380-021-01342-4 (PMC8515782; doi:10.1038/s41380-021-01342-4)
Supplement: Supplementary file 2 — Supplementary Methods [file 41380_2021_1342_MOESM2_ESM.pdf]

Supplementary Methods

Study design

The study was designed for unbiased exploration of the transcriptomic and proteomic changes that occur in the MeA of adult male rats following acute social isolation and in several time points after the isolated animals were returned to group housing, as depicted in Fig. 1C

The RNA-Seq analysis (Exp-1) included 48 rats, with each sample containing a pool of bilateral MeA punches taken from two rats, in order to obtain a sufficient amount of mRNA for the RNA-Seq analysis. We evaluated the RNA-Seq results on selected genes using qPCR on the same samples (Supplementary Data file 1), as well as two more independent sample sets (Exp-2, Exp-3) identical to the first one, except that they included only one animal per sample (Supplementary Data file 2). Finally, we generated another sample set for the proteomic analysis, which was similar to the previous ones, with one animal per sample (Supplementary Data file 3). We also added to this set a group of animals sampled after four hours of regrouping (ReGr 4h). This was done because we reasoned that proteomic changes at four hours might reflect the transcriptomic changes that occur two hours after regrouping.

Animals

The animals were housed in the SPF animal facility of the University of Haifa under veterinary supervision. Following arrival to the animal facility, the animals had a minimum of one-week habituation prior to the experiment. Group housed animals were kept in groups of 2-6 animals within 60 x 40 x 20 cm cages. For isolation, animals were singly housed in 50 x 30 x 20 cm cages for seven days. For regrouping, isolated animals were housed with their original cage-mates in groups of 2-4 within 50 x 30 x 20 cm cages for 2, 4 or 24 hours prior to decapitation. Grouped, isolated and regrouped animals were all kept together in the same room.

Behavioral experiments

Social recognition memory (SRM)

Prior to the behavioral tests, adult subjects were transferred to a fresh cage (50 x 30 x 20 cm) in a separate room with dim red light and good ventilation for a 1 hour of habituation. Up to 10 cages were arranged, side-by-side, at least 10 cm apart. The SRM test was conducted as previously described [1]. Briefly, a 5-min encounter of the subject with a novel juvenile (E1) was followed two hours later by a second 5-min encounter (E2) with the same juvenile. At the end of each encounter, the juvenile was removed from the test room. During each encounter, the duration of social investigation, including any contact between the subject's nose and the juvenile's body or any investigatory behavior, such as following, displayed by the adult subject was measured using a stopwatch.

Social novelty preference (SNP)

SNP experiments were carried out as previously described [2]. Briefly, habituation, sampling and testing were all carried out in a black Plexiglas arena (45 x 45 x 43 cm) and stimuli were placed in transparent Plexiglas corrals (9 cm in diameter, slotted with five horizontal 0.5 x 13.5 cm slots per side, to allow physical contact between the animals, and covered with transparent Plexiglas to prevent escape). One day prior to the sampling phase, the rat subjects were habituated in the arena (A black Plexiglas arena 45 x 45 x 43 cm) with the corrals for 10 minutes. During the sampling phase, each rat subject was exposed to both an unfamiliar juvenile rat and an object that were placed within two corrals at two opposite corners of the arena for 1 hour, after which the rat was returned to its home-cage. One day later, the same subject was placed again in the arena (testing phase) for 5 min, together with the familiar juvenile from the day before and a novel juvenile rat, located within two corrals at two opposite corners of the arena. The test was videotaped and the time each subject spent investigating each corral was measured with a stopwatch by an experimenter blind to the animal's condition (G vs. Iso 7d).

Quantitative PCR analysis

A melting curve protocol was included in each run to verify amplification specificity. *Gapdh* and *Hprt* were selected as reference housekeeping genes for internal normalization of gene expression. Levels were compared to the average of the two normalizing genes and to the G1 sample in each experiment. Real-time PCR results were

analyzed using StepOne software (Applied Biosystems) with ΔΔCT protocol. Next, average relative quantification (RQ) values were calculated for each of the three experiments and compared among the four experimental groups across the three independent biological replicates. As cases where normality or homogeneity of variance were not satisfactory, we performed all comparisons by Kruskal-Wallis test, followed by Dunn's post hoc tests. Differences were considered significant for *P* value <0.05. Pearson's correlation (calculated and tested using R 'rcorr' function) was used to determine correlation between RT-PCR and RNA-Seq, and between RT-PCR and proteomics.

Proteomic analysis

Raw proteomic data was processed with MaxQuant v1.6.0.16, searched with the Andromeda search engine against the rat protein database and appended with common laboratory protein contaminants and the following modifications: fixed modification- cysteine carbamidomethylation; variable modifications- methionine oxidation; asparagine and glutamine deamidation; protein N-terminal acetylation. Decoy hits were filtered out, as well as proteins that were identified on the of basis one peptide only. Intensities indicated per protein detected per sample were log2 transformed. We have achieved successful proteomics sequencing for only four out of six replicates from the Isolated condition. Therefore, we selected an equal number of samples from all of the other groups by calculating pair-wise Euclidean distances between log2 transformed protein expression data. The quantitative comparisons were calculated using Perseus v1.6.0.7. Pair-wise t-test with Weltch correction were calculated. Differences were considered significant for *P* value <0.05 and fold change >1.3.

References

1. Shahar-Gold, H., R. Gur, and S. Wagner, *Rapid and reversible impairments of short- and long-term social recognition memory are caused by acute isolation of adult rats via distinct mechanisms.* PLoS One, 2013. **8**(5): p. e65085.
2. Gur, R., A. Tendler, and S. Wagner, *Long-term social recognition memory is mediated by oxytocin-dependent synaptic plasticity in the medial amygdala*. Biol Psychiatry, 2014. **76**(5): p. 377-86.
