# Supplementary material for: Acute social isolation and regrouping cause short- and long-term molecular changes in the rat medial amygdala
Source: Mol Psychiatry. 2021 Oct 14;27(2):886–95. doi: 10.1038/s41380-021-01342-4 (PMC8515782; doi:10.1038/s41380-021-01342-4)
Supplement: Supplementary file 3 — Supplementary Figures [file 41380_2021_1342_MOESM3_ESM.pdf]

Supplementary Figures

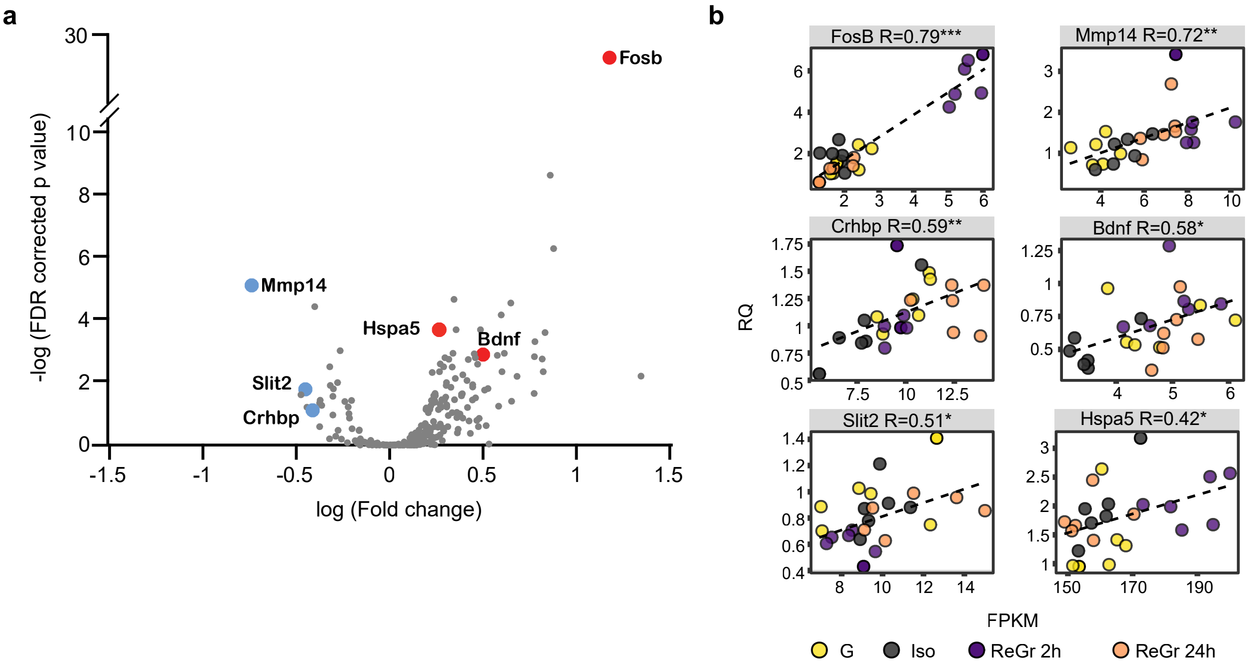

Supplementary Figure 1. Validation of RNA-seq results by qPCR in selected genes.

a) Volcano plot of the 119 DE genes in the ReGr 2h animals vs. Iso 7d and ReGr 24h. Genes that were selected for validation are marked according to the direction of change in the transcriptome: downregulated (Blue) and upregulated (Red).

b) Real-time qPCR verification of the six selected genes in the same samples used for RNA-Seq, color-coded according to the experimental group. Spearman's correlation between relative quantification (RQ) values of the qPCR analysis and FPKM values of the RNA-Seq analysis was calculated and tested for significance. The name of examined gene, the correlation R value and the significance level are denoted above each correlation. *** p<0.001; ** p<0.01; * p<0.05.

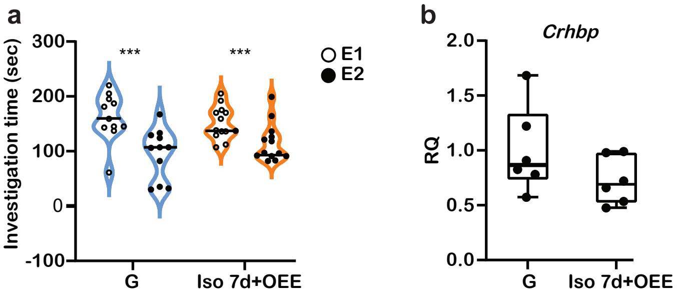

Supplementary Figure 2. Odor-enriched environment (OEE) rescues SRM is isolated animals.

a) Social recognition memory (SRM), demonstrated by a reduction in investigation time between two consecutive 5-min encounters (E1, E2) of the subject rat with the same social stimulus, using a 120 min inter-encounter interval. Note that the Iso 7d animals exhibit intact SRM despite isolation, when exposed to OEE. ***p<0.001, paired t-test following main effect in 2-way repeated ANOVA. Horizontal lines represent median values.

b) Normalized RQ values of Crhbp mRNA levels in the MeA of G and Iso 7d+OEE (top). No significant changes are observed, suggesting that environment enrichment restores reverses the effect of isolation of the expression of Crhbp.

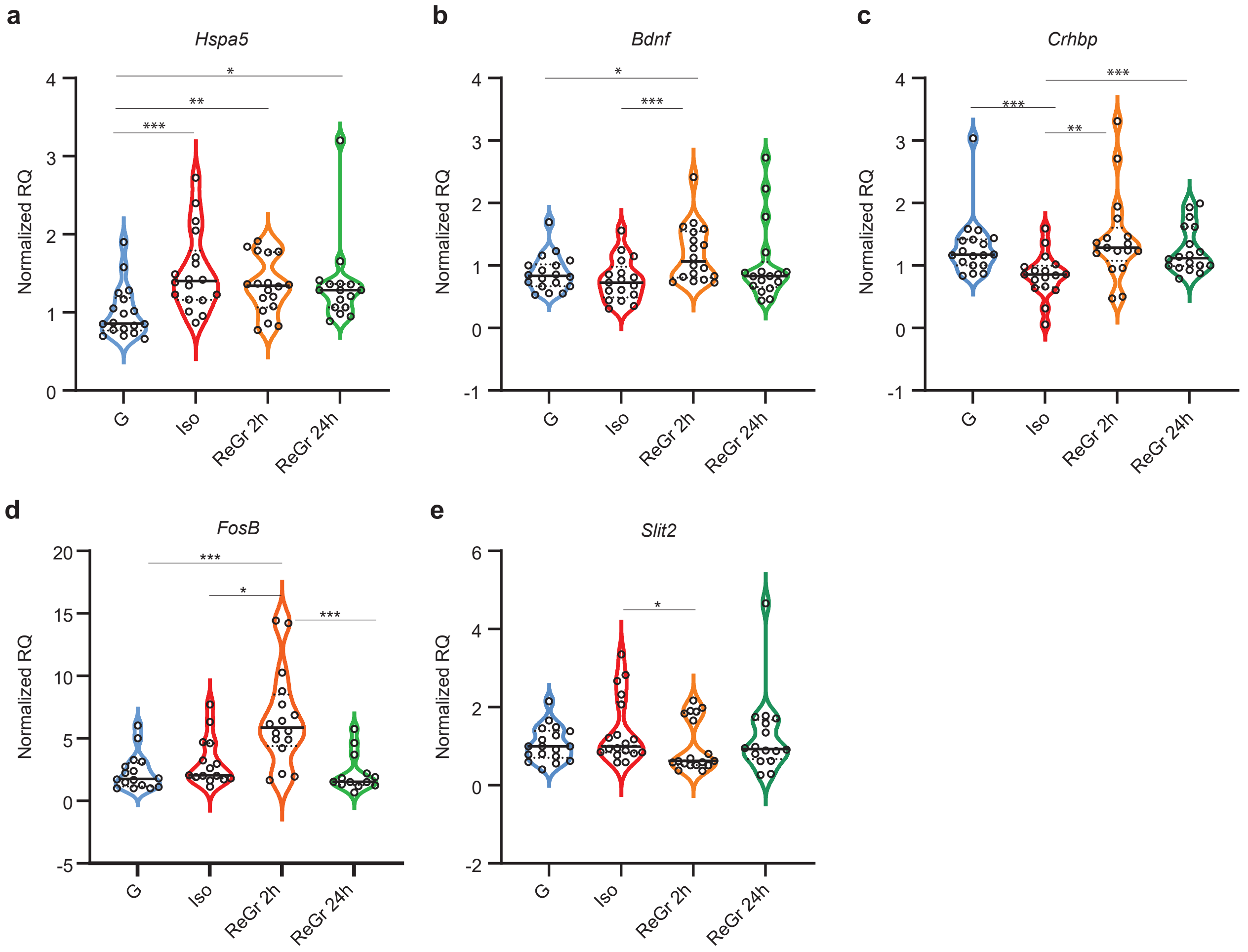

Supplementary Figure 3. Real-time qPCR validation of transcriptomic changes in five genes, in the three independent MeA sample sets.

Normalized RQ values of qPCR analysis of the combined samples from the three independent sample sets (Exp-1,2,3,) for five genes. *P<0.05, **P<0.01, ***P<0.001, Dunn's post hoc tests following main effect in Kruskal-Wallis test.
